# Alkaline Water and Longevity: A Murine Study

**DOI:** 10.1155/2016/3084126

**Published:** 2016-05-31

**Authors:** Massimiliano Magro, Livio Corain, Silvia Ferro, Davide Baratella, Emanuela Bonaiuto, Milo Terzo, Vittorino Corraducci, Luigi Salmaso, Fabio Vianello

**Affiliations:** ^1^Department of Comparative Biomedicine and Food Science, University of Padua, 35020 Legnaro, Italy; ^2^Department of Management and Engineering, University of Padua, 36100 Vicenza, Italy

## Abstract

The biological effect of alkaline water consumption is object of controversy. The present paper presents a 3-year survival study on a population of 150 mice, and the data were analyzed with accelerated failure time (AFT) model. Starting from the second year of life, nonparametric survival plots suggest that mice watered with alkaline water showed a better survival than control mice. Interestingly, statistical analysis revealed that alkaline water provides higher longevity in terms of “deceleration aging factor” as it increases the survival functions when compared with control group; namely, animals belonging to the population treated with alkaline water resulted in a longer lifespan. Histological examination of mice kidneys, intestine, heart, liver, and brain revealed that no significant differences emerged among the three groups indicating that no specific pathology resulted correlated with the consumption of alkaline water. These results provide an informative and quantitative summary of survival data as a function of watering with alkaline water of long-lived mouse models.

## 1. Introduction 

Alkaline water, often referred to as alkaline ionized water (AKW), is commercially available and is mainly proposed for electrolyte supplementation during intensive perspiration. Early studies on animal models reported that alkaline water supplementation may exert positive effects on body weight improvement and development in offspring [1, 2]. Even biochemical markers were analyzed, suggesting that alkaline ionized water intake can cause elevation of metabolic activity. In particular, hyperkaliemia was observed in 15-week-old rats and pathological changes of necrosis in myocardial muscle were found [3].

More recently, studies were carried out on alkaline reduced water (ARW), referring to electrolyzed water produced from minerals, such as magnesium and calcium, which is characterized by supersaturated hydrogen, high pH, and a negative redox potential. This hydrogen-rich functional water has been introduced as a therapeutic strategy for health promotion and disease prevention [4].

Alkaline and electrolyzed water have been shown to exert a suppressive effect on free radical levels in living organisms, thereby resulting in disease prevention [5]. Various biological effects, such as antidiabetic and antioxidant actions [4], DNA protecting effects [6], and growth-stimulation activities [2], were documented.

Although a variety of bioactive functions have been reported, the effect of alkaline water on lifespan and longevity in vivo is still unknown. Animal alkalization has been shown to be well tolerated and to increase tumor response to metronomic chemotherapy as well the quality of life in pets with advanced cancer [7]. Therefore, we performed a study based on survival rate experiments, which play central role in aging research and are generally performed to evaluate whether specific interventions may alter the aging process and lifespan in animal models.

## 2. Materials and Methods 

Biological effects of alkaline water were evaluated on a selected population of 150 mice (CD1, by Charles River, Oxford, UK). Pathogen-free mice were purchased and placed in a specific breeding facility. No other animal was present in the room. Contact with animal caretakers was minimized to feeding and watering. The population was divided into 3 groups, each consisting of 50 individuals, as follows:

- Group A: 50 mice conventionally fed and watered with alkaline water produced by the Water Ionizer (mod. NT010) by Asiagem (Italy). The Water Ionizer is a home treatment device for producing alkaline drinking water.
- Group B: 50 mice conventionally fed and watered with alkalized water obtained by dilution of a concentrated alkaline solution (AlkaWater by Asiagem, Italy). AlkaWater is a concentrated alkaline solution for preparing alkaline drinking water.
- Group C: 50 mice conventionally fed and watered as conventional (control group) with tap water. The local water supply was evaluated weekly for assuring the absence of toxins and pathogens. The pH values were in the 6.0–6.5 range.

All procedures involving animals were conducted in accordance with the Italian law on experimental animals and were approved by the Ethical Committee for Animal Experiments of the University of Padua and the Italian health Ministry (Aut. no. 39ter/2011). Efforts were made to minimize animal suffering.

### 2.1. Histological Examination

Treated aged mice were sampled postmortem and subjected to histological examination. Animals belonging to the populations treated with alkaline water, A and B, were sacrificed after 24 months and compared to mice treated with tap water. Samples from kidneys, intestine, heart, liver, and brain were fixed in 10% neutral buffered formalin, and 4 *μ*m sections were analyzed by optical microscopy.

### 2.2. Statistical Analysis

In order to investigate the biological influence of alkaline water on mouse longevity, we employed the accelerated failure time model (AFT) [8], which allows formally exploring the possible effect on survival curves of the applied three-level treatment, that is, examining the role of group membership as a covariate of lifespan. As a more robust alternative to the commonly used proportional hazards models, such as the Cox model, the use of AFT models is advised in the field of survival analysis when the goal is to investigate if a covariate may affect the lifespan in a way that the life cycle may pass more or less rapidly. In fact, whereas a proportional hazard model assumes that the effect of a covariate is constant over time, an AFT model assumes that the effect of a covariate is to accelerate or decelerate the life course.

The relevance of AFT model for biomedical studies has been already recognized in the literature [8]. With more specific reference to the issue of aging, Swindell [9] observed that some genetic manipulations were found to have a multiplicative effect on survivorship which were well characterized by the AFT model “deceleration factor.” Moreover, Swindell [9] argued also that the AFT model should be utilized more widely in aging research since it provides useful tools to maximize the insight obtained from experimental studies of mouse survivorship.

To perform all calculations, we applied a parametric survival analysis approach using a class of 3-parameter AFT distribution models implemented within the statistical software Minitab, version 17.2.1 [10]. More specifically, we employed three types of random distributions, namely, log-logistic, log-normal, and generalized Weibull.

## 3. Results 

The experiment consisted in an initial 15-day acclimatization period. After acclimatization, animals (50, group A) were watered with alkaline water (pH 8.5), obtained by the Water Ionizer (Asiagem, Italy), whereas group B animals (50) were watered with water alkalized at pH 8.5 by a concentrated alkaline solution (AlkaWater by Asiagem, Italy) for 15 days. Group C animals (50), control group, were watered with the local water supply. This period has been identified to gradually accustom the animals treated with alkaline water. At the end of the second period of acclimatization, group A and B animals were watered with alkaline water at pH 9.5 (by the Water Ionizer and by AlkaWater by Asiagem, Italy), while animals of group C were watered with local tap water.

After the first year, the most aggressive individuals were moved to other cages within the same group and an environmental enrichment protocol was employed in order to decrease the hyperactivity. This phenomenon was observed especially in animals of groups A and B.

Table 1 reported basic statistics on mice survival of treated and control animals.

Regarding group A, animals (50) were watered with alkaline water (pH 8.5), obtained by the Water Ionizer (Asiagem, Italy). As for group B, animals (50) were watered with water alkalized at pH 8.5 by a concentrated alkaline solution (AlkaWater by Asiagem, Italy) for 15 days. Regarding group C, animals (50), control group, were watered with the local water supply.

**Figure 1:**
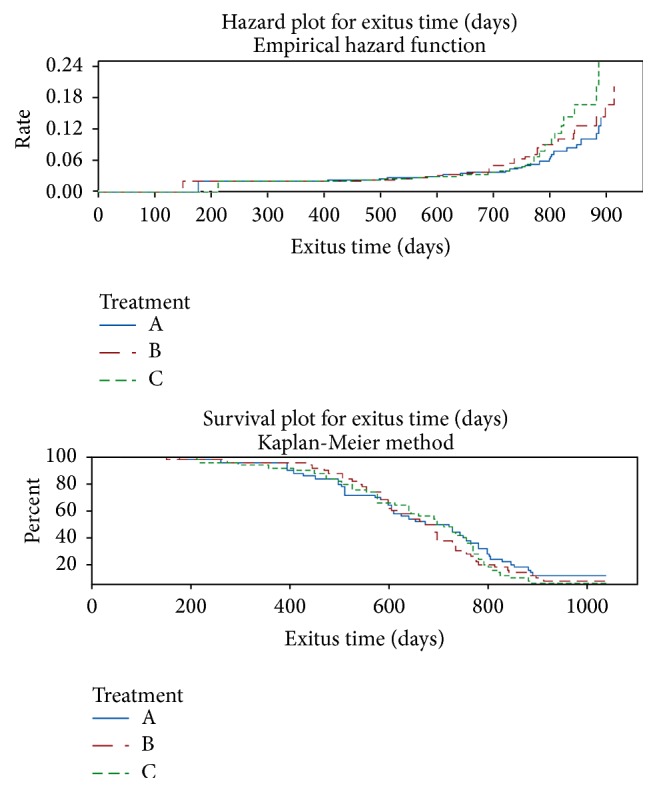
Nonparametric hazard and survival plots by treatment level. Group A: animals (50) were watered with alkaline water (pH 8.5), obtained by the Water Ionizer (Asiagem, Italy). Group B: animals (50) were watered with water alkalized at pH 8.5 by a concentrated alkaline solution (AlkaWater by Asiagem, Italy) for 15 days. Group C: animals (50), control group, were watered with the local water supply.

A first look on experimental data is provided in Figure 1, where nonparametric hazard and survival plots seem to suggest that even if no macroscopic difference emerges, starting from the second year of life mice watered with alkaline Water Ionizer and those treated with AlkaWater overwhelmed control mice.

**Figure 2:**
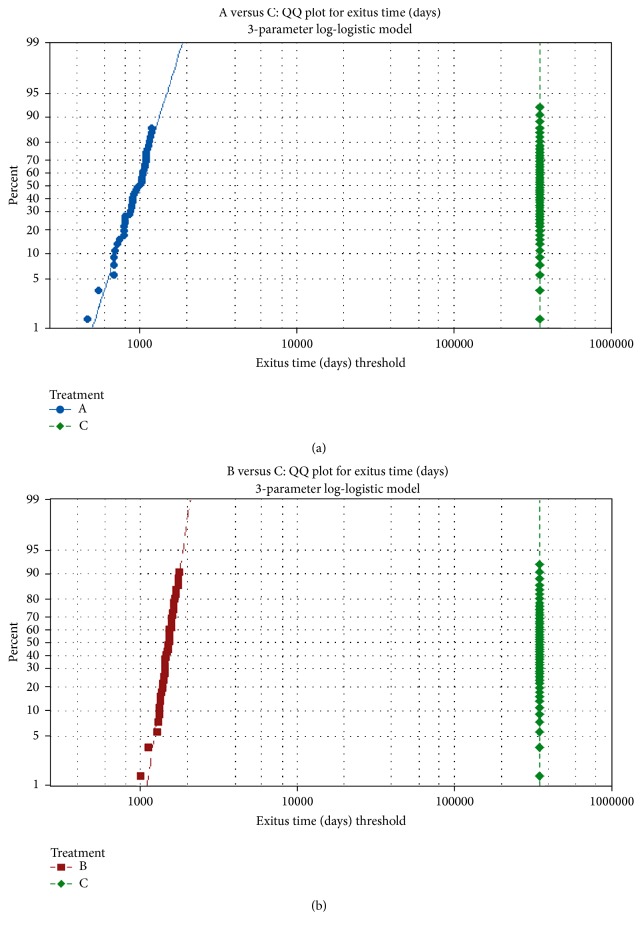
QQ plots using the 3-parameter log-logistic distribution model. (a) Treatment A survival time quantiles (vertical axis) versus treatment C survival time quantiles (horizontal axis); (b) treatment B survival time quantiles (vertical axis) versus treatment C survival time quantiles (horizontal axis).

In order to explore the possible effect of different treatments, that is, to examine the role of group membership on longevity, we applied a parametric survival analysis approach using a class of 3-parameter survival distributions that represent flexible accelerated failure time, AFT models. First of all, using the Anderson-Darling goodness-of-fit statistic, we compared three specific survival distributions, that is, log-logistic (AD = 6.397), log-normal (AD = 6.519), and generalized Weibull (AD = 6.447). Since the best fitting was shown by log-logistic model, we adopted this one as final survival distribution model. The straight lines in the log-logistic distribution QQ plots (Figures 2(a) and 2(b)) indicate that this distribution provides a suitable fit to our survival data.

**Figure 3:**
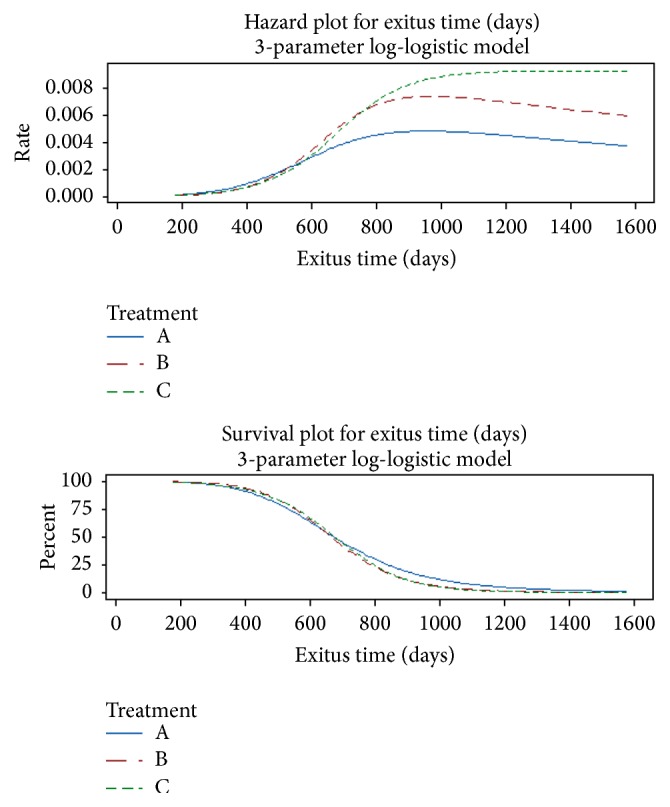
Distribution plot results using the 3-parameter log-logistic model. Group A: animals (50) were watered with alkaline water (pH 8.5), obtained by the Water Ionizer (Asiagem, Italy). Group B: animals (50) were watered with water alkalized at pH 8.5 by a concentrated alkaline solution (AlkaWater by Asiagem, Italy) for 15 days. Group C: animals (50), control group, were watered with the local water supply.

Finally, by including our treatment as covariate, we performed a parametric distribution analysis whose results are graphically represented in Figure 3.

Starting with the second year of life, it is worth noting that both alkaline water treated groups denote a decreasing hazard curve over time, while the corresponding curve for control group is monotonically increasing. To more formally compare the treatment levels, the proposed analysis provided also suitable *p* values. Since the *p* values related on the null hypotheses of equality of location, scale and threshold parameters were, respectively, less than 0.001 (for both locations and scales) and 0.634 (for thresholds) at a 5% significance level; we can state that there is enough experimental evidence to conclude that the treatment significantly affects the mice longevity; in particular the alkaline water provides a benefit to longevity in terms of “deceleration aging factor” as it decreases the hazard functions when compared with the control group. Note that the treatment effect cannot be directly related to no one of the three distribution parameters. Anyway, using the estimated parameters, it should be possible to provide an estimate for the effect of each treatment on survivorship: setting the reference survival time to 1000, 1200, and 1400 days, Table 2 summarizes the estimated point and 95% interval survival probabilities by each treatment level.

As final remark, it should be noted that even if our parametric AFT survival analysis was performed using the log-logistic distribution, our conclusions are consistent with results obtained using the generalized Weibull distribution, while via log-normal distribution no significant effect was found.

### 3.1. Histological Examination

**Figure 4:**
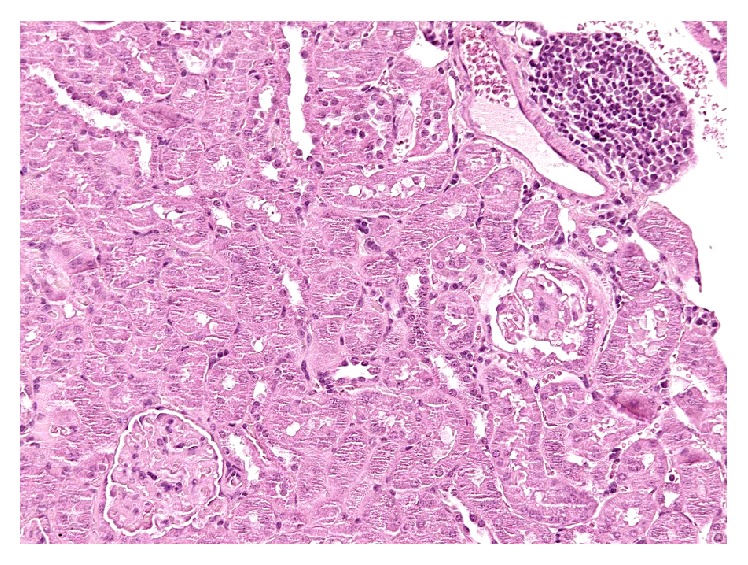
Kidney, a specific chronic nephropathy. Focal interstitial mainly lymphocytic infiltrate (upright) and a sclerotic glomerulus (middle right). Hematoxylin and Eosin.

No significant differences emerged from the histological examination among the three groups. In all examined samples, renal tissue was characterized by a mild-to-moderate lymphoplasmacytic interstitial infiltrate and few occasional glomerular changes as glomerular size reduction and increasing of Bowman's space (Figure 4).

Final diagnosis was mild chronic progressive nephropathy for the three analyzed mouse groups.

**Figure 5:**
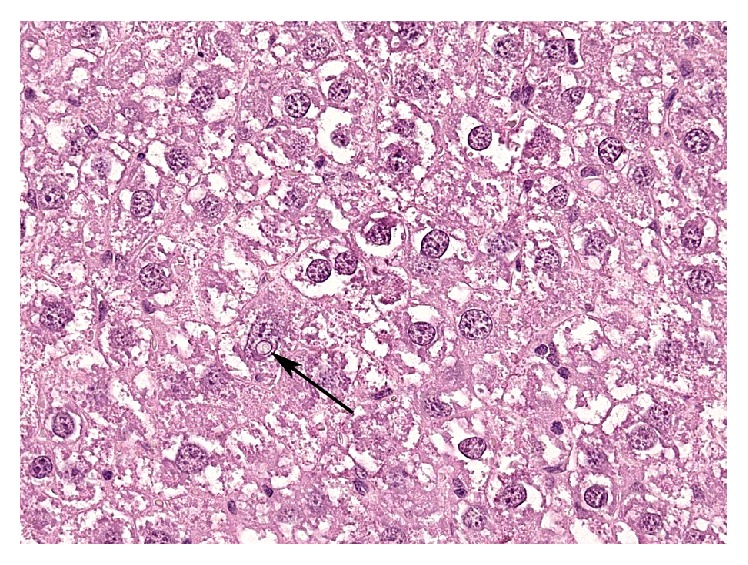
Liver, aging change. Hepatocellular abundant dishomogeneous cytoplasm, binucleation (center), variably sized nuclei, and a nuclear pseudoinclusion cyst (arrow). Hematoxylin and Eosin.

The microscopic examination of the liver revealed a multifocal nodular pattern of the parenchyma and diffuse mild-to-moderate hepatocellular cytoplasmic hydropic degeneration with multifocal binucleation in all explored animals (Figure 5).

Mild-to-moderate anisokaryosis was the most relevant alteration, with few pleomorphic nuclei and frequent intranuclear pseudoinclusions and karyomegaly. A specific mild perivascular infiltrate was occasionally present. Final diagnosis was mild-to-moderate diffuse hepatopathy with multifocal hyperplastic hyperplasia.

**Figure 6:**
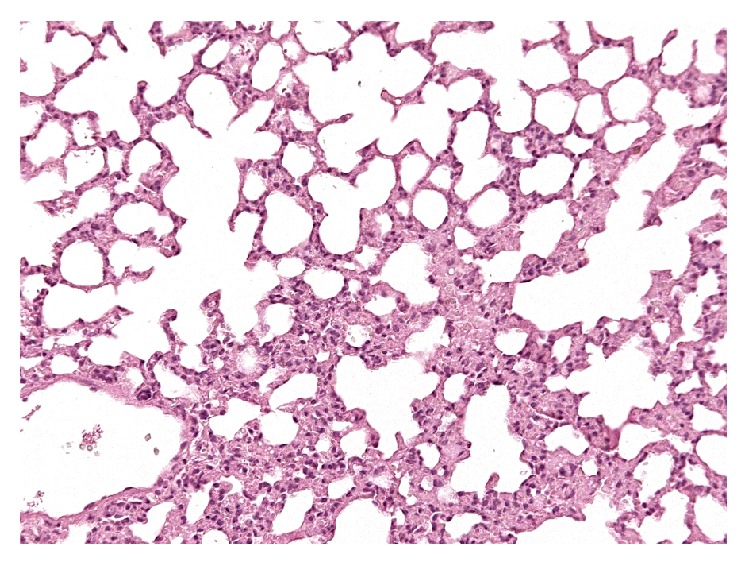
Lung, mild atelectasis. Very mild multifocal interstitial thickening of the alveolar septa associated with congestion and mild cellular increase. Hematoxylin and Eosin.

The pulmonary parenchyma showed mild multifocal areas of interstitial thickening of the interalveolar septa due to moderate congestion and mild cellular mixed infiltrate (Figure 6). Mild areas of emphysema were detected at the periphery of the parenchyma. Final diagnosis was multifocal very mild atelectasis and mild vicarious emphysema.

**Figure 7:**
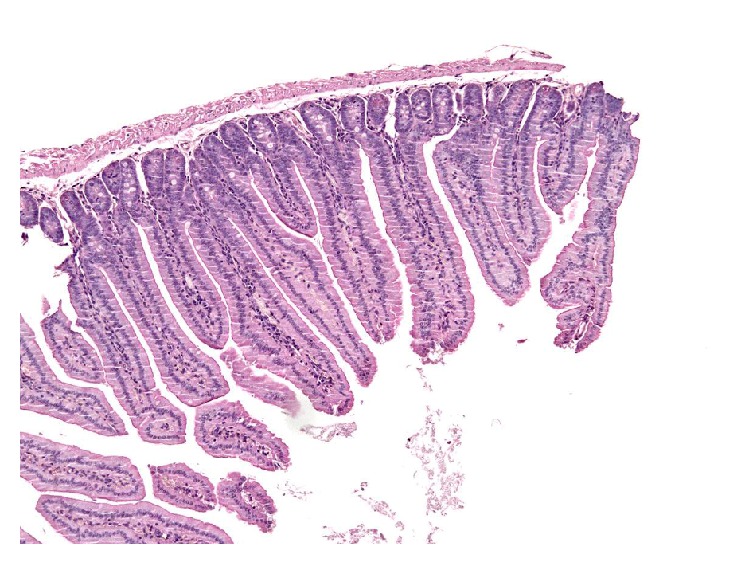
Intestine. Longitudinal section of duodenum showing uniformly thin and elongated villi. Hematoxylin and Eosin.

At the same time, no relevant histopathologic histological changes have been noticed in intestine (Figure 7), brain, and heart.

## 4. Discussion

The present work presents a 3-year survival study on a population of 150 mice and the data were analyzed with accelerated failure time (AFT) model. Kaplan-Meier statistical analysis of the survival data indicates the possibility of a positive effect of alkaline water on mouse lifespan and AFT model allowed evaluating differences starting from the second year of the survival curves. These results provide an informative and quantitative summary of survival data as a function of watering with alkaline water on long-lived mouse models. It should be pointed out that, from the standpoint of aging research, this statistical approach presents appealing properties and provides valuable tools for the analysis of survival. The observation of tissues of deceased animals was performed for the assessment of the state of internal organs to be compared with similar analyses of untreated animals. The renal lesions observed at histology were specific and common for the three animal groups. Chronic progressive nephropathy has been well described as normal aging change in mice [11, 12]. In our cases animals did not show any clinical sign of nephropathy or any other histological evidence of specific kidney disease and we ascribed the lesions to the aging process [11, 12].

The examined livers were also affected by typical lesions of mature subjects, such as hyperplastic nodules. Furthermore, well known aging changes were individuated in the hepatocytes, such as karyomegaly, nuclear pleomorphism, and pseudoinclusions cysts [11, 12].

## 5. Conclusions

A 3-year survival study on a population of 150 mice was carried out in order to investigate the biological effect of alkaline water consumption. Firstly, nonparametric hazard and survival plots suggest that mice watered with alkaline water overwhelmed control mice. Secondly, data were analyzed with accelerated failure time (AFT) model inferring that a benefit on longevity, in terms of “deceleration aging factor,” was correlated with the consumption of alkaline water. Finally, histological examination of mice kidneys, intestines, hearts, livers, and brains was performed in order to verify the risk of diseases correlated to alkaline watering. No significant damage, but aging changes, emerged; organs of alkaline watered animals resulted to be quite superimposable to controls, shedding a further light in the debate on alkaline water consumption in humans.

## Figures and Tables

**Table 1:** Basic statistics on mice survival by treatment level.

Treatment level	Mortality rate %	Lifespan mean (std. dev.) Days
Group A	88	679 (209)
Group B	92	671 (180)
Group C	96	667 (185)

**Table 2:** Table of survival probabilities by treatment level. The probabilities, along with their related 95% confidence interval limits, were calculated using the normal approximation.

Treatment level	Time (days)	Estimated probability	Lower 95% CI limit	Upper 95% CI limit
A	1000	0.116	0.056	0.226
	1200	0.046	0.014	0.140
	1400	0.020	0.004	0.098

B	1000	0.055	0.021	0.137
	1200	0.013	0.003	0.066
	1400	0.004	0.000	0.039

C	1000	0.049	0.022	0.106
	1200	0.008	0.002	0.027
	1400	0.001	0.000	0.007
